# Specific microRNA Profile Associated with Inflammation and Lipid Metabolism for Stratifying Allergic Asthma Severity

**DOI:** 10.3390/ijms25179425

**Published:** 2024-08-30

**Authors:** Andrea Escolar-Peña, María Isabel Delgado-Dolset, Carmela Pablo-Torres, Carlos Tarin, Leticia Mera-Berriatua, María del Pilar Cuesta Apausa, Heleia González Cuervo, Rinku Sharma, Alvin T. Kho, Kelan G. Tantisira, Michael J. McGeachie, Rocio Rebollido-Rios, Domingo Barber, Teresa Carrillo, Elena Izquierdo, María M. Escribese

**Affiliations:** 1 Department of Basic Medical Sciences, Institute for Applied Molecular Medicine Nemesio Díez, School of Medicine, Universidad San Pablo-CEU, CEU Universities, Urbanización Montepríncipe, 28668 Boadilla del Monte, Spain; andrea.escolarpena@ceu.es (A.E.-P.); maria.delgadodolset@ceu.es (M.I.D.-D.); carmela.pablojimenez@ceu.es (C.P.-T.); leti.mera@gmail.com (L.M.-B.); domingo.barberhernandez@ceu.es (D.B.); 2 R+D Department, Atrys Health, 08025 Madrid, Spain; ctarin@atryshealth.com; 3 Allergy Service, Hospital Universitario de Gran Canaria Doctor Negrín, 35010 Las Palmas de Gran Canaria, Spain; pili_cuesta@hotmail.com (M.d.P.C.A.); heleia.gc@gmail.com (H.G.C.); teresa.carrillo@ulpgc.es (T.C.); 4 Department of Medicine, Channing Division of Network Medicine, Brigham and Women’s Hospital, Harvard Medical School, Boston, MA 02115, USA; rersh@channing.harvard.edu (R.S.); remmg@channing.harvard.edu (M.J.M.); 5 Computational Health Informatics Program, Boston Children’s Hospital, Boston, MA 02115, USA; alvin_kho@hms.harvard.edu; 6 Division of Pediatric Respiratory Medicine, University of California San Diego and Rady Children’s Hospital, San Diego, CA 92123, USA; ktantisira@health.ucsd.edu; 7 Department I of Internal Medicine, Centre of Integrated Oncology Aachen Bonn Cologne Duesseldorf (CIO ABCD), Faculty of Medicine and University Hospital of Cologne, University of Cologne, 50923 Cologne, Germany; rocio.rebollido-rios@uk-koeln.de; 8 Center for Molecular Medicine Cologne, University of Cologne, 50923 Cologne, Germany; 9 CECAD Center of Excellence on Cellular Stress Responses in Aging-Associated Diseases, University of Cologne, 50923 Cologne, Germany

**Keywords:** biomarkers, miRNAs, allergic asthma, severity

## Abstract

The mechanisms underlying severe allergic asthma are complex and unknown, meaning it is a challenge to provide the most appropriate treatment. This study aimed to identify novel biomarkers for stratifying allergic asthmatic patients according to severity, and to uncover the biological mechanisms that lead to the development of the severe uncontrolled phenotype. By using miRNA PCR panels, we analyzed the expression of 752 miRNAs in serum samples from control subjects (*n* = 15) and mild (*n* = 11) and severe uncontrolled (*n* = 10) allergic asthmatic patients. We identified 40 differentially expressed miRNAs between severe uncontrolled and mild allergic asthmatic patients. Functional enrichment analysis revealed signatures related to inflammation, angiogenesis, lipid metabolism and mRNA regulation. A random forest classifier trained with DE miRNAs achieved a high accuracy of 97% for severe uncontrolled patient stratification. Validation of the identified biomarkers was performed on a subset of allergic asthmatic patients from the CAMP cohort at Brigham and Women’s Hospital, Harvard Medical School. Four of these miRNAs (hsa-miR-99b-5p, hsa-miR-451a, hsa-miR-326 and hsa-miR-505-3p) were validated, pointing towards their potential as biomarkers for stratifying allergic asthmatic patients by severity and providing insights into severe uncontrolled asthma molecular pathways.

## 1. Introduction

Asthma is a multifactorial, chronic syndrome that involves genetic and environmental factors [1]. According to World Health Organization (WHO) estimates, approximately 262 million people worldwide suffered from asthma in 2019 [2], highlighting the significant global burden of this disease. Asthma not only affects the physical health of individuals but also impacts their quality of life, work productivity and healthcare utilization, resulting in substantial economic and social costs. This condition causes an elevated number of deaths globally every year (461.07 thousand of deaths in 2019) [3], and its symptoms and intensity may change over time [4]. The hallmarks of asthma include chronic airway inflammation, airway hyperresponsiveness and airway remodeling [3]. Airway remodeling involves structural changes in the airway walls, which contribute to the narrowing of the airways and persistent airflow limitation [3], exacerbating the severity of the disease.

Allergic asthma is the most predominant among the multiple asthma phenotypes [5]. It is defined by a sensitization to environmental allergens and its subsequent clinical response; specifically, around 85% of allergic asthma patients present house dust mite (HDM) sensitization [5,6]. As previously described, allergic asthma is characterized by significant airway remodeling, which begins with the disruption of the epithelial barrier due to exposure to allergens, such as HDMs. Key structural changes during airway remodeling include loss of barrier integrity, goblet cell metaplasia (the hypersecretion of mucus), airway smooth muscle hyperplasia and subepithelial fibrosis. The respiratory epithelium plays a crucial role in maintaining airway integrity. In response to allergens, proteases can damage tight junctions, facilitating further inflammation and contributing to the chronic nature of asthma [1]. The immune response in allergic asthma is predominantly mediated by Th2 cells, which produce cytokines such as IL-4, IL-5, and IL-13, driving the allergic inflammatory process. Dendritic cells (DCs) play a pivotal role in presenting allergens to T cells, promoting Th2 differentiation. Additionally, eosinophils and mast cells are essential to the pathophysiology of asthma, with eosinophilia serving as a marker of disease severity. Mast cells release mediators that enhance airway hyperresponsiveness and contribute to remodeling [1]. Moreover, allergic asthma development is influenced by gene–environment interactions. These include variants in Filaggrin, ORMDL3 (ORM (yeast)-like protein isoform 3), TSLP (Thymic Stromal Lymphopoietin) promoter genes and the chromosome 17q12-21 locus. Additionally, polymorphisms in the IL-4 receptor alpha chain and DNA methylation changes in the nasal epithelia have been associated with allergic asthma susceptibility [7].

Among asthmatic patients, there is a progression in severity that depends on different factors such as years of evolution, comorbidities or lack of control of the disease. There is a specific phenotype that remains uncontrolled despite adherence to maximally optimized therapy and contributing factors. Patients with this phenotype are considered severe uncontrolled allergic asthmatic patients; they are classified in GINA steps 4–5, which are part of the Global Initiative for Asthma (GINA [8]) classification system. This system categorizes asthma severity and guides treatment based on symptom control and risk factors, ranging from step 1 (mild asthma) to step 5 (severe asthma).

On the one hand, mild allergic asthmatic patients (GINA steps 1–3) typically experience well-controlled symptoms, less frequent use of rescue medication and relatively normal or slightly reduced lung function. These patients generally experience fewer exacerbations and less impact on daily activities. Their treatment usually involves low to medium doses of inhaled corticosteroids (ICSs), sometimes combined with long-acting beta-agonists (LABAs) or leukotriene receptor antagonists (LTRAs). On the other hand, severe uncontrolled allergic asthmatic patients (GINA 4–5) display poor symptom control (frequent symptoms or reliever use, activity limitations, night waking, etc.) and/or frequent (≥2/year, requiring oral corticosteroids, OCS, to control) or serious (≥1/year requiring hospitalization) exacerbations, all this resulting in a poor quality of life for these patients [9]. Severe uncontrolled and persistent inflammation, along with the airway remodeling associated with this condition, can lead to irreversible airflow obstruction and respiratory failure, ultimately resulting in death in some cases. This risk is particularly pronounced in patients with severe uncontrolled asthma, who often require expensive treatments. The burden of asthma, including premature death, remains especially high in countries with a low socio-demographic index, where access to advanced treatments may be limited [3].

The identification of these patients is currently a priority, to provide them with the most appropriate treatment possible in the first place, to ensure quality of life improvement and to reduce the health system economic burden. This paper proposes a comprehensive strategy for the identification of biomarkers by comparing distinct allergic asthmatic phenotypes with differentiating clinical variables according to their severity: mild vs. severe uncontrolled.

MicroRNAs (miRNAs) are being widely studied as disease biomarkers in diseases such as cancer, cardiovascular diseases, sepsis or nervous system disorders [10], as well as allergic diseases and asthma [11,12,13,14].

miRNAs are small, non-coding RNAs which regulate one third of the organism coding genes [15]. Thus, miRNAs can modulate almost all biological processes, being essential for maintaining cellular homeostasis. In recent years, several studies have also been focused on investigating the role of miRNAs in the development of asthma [10,12]. Likewise, it has been demonstrated that miRNAs can modify the function of different cell types leading to asthmatic inflammation [12] by influencing the secretion of cytokines and chemokines, which affect smooth muscle cells’ homeostasis, airway remodeling and hyperreactivity [16,17]. On the other hand, miRNAs have also been described as regulating the cell proliferation process in eosinophilic asthma by controlling the expression of IL-13, IL-1β and CCL11 [18] and lymphocytes’ and eosinophils’ functions, as well as influencing the release of TH2 cytokines and M1/M2 macrophage polarization [12].

Several studies have identified miRNAs associated with asthma, such as miR-21 and miR-223 [19]. However, these studies did not account for asthma severity. Given the established role of miRNAs in asthma, this study aims to identify miRNAs as potential biomarkers for allergic asthma severity by comparing groups belonging to a well-defined cohort of mild and severe uncontrolled allergic asthmatic patients.

## 2. Results

### 2.1. Subject Classification in Study Cohort

The clinical history of the subjects was thoroughly analyzed as previously described [20]. There were no statistical differences related to sex, onset age, BMI, smoking status or total IgE levels (*p*-value > 0.05). 

Regarding the limitations of this study, age was found to be significantly higher in the severe uncontrolled group (50.90 ± 10.62 years) compared to the mild (36.00 ± 6.53 years) and control (37.40 ± 13.95 years) groups (Table 1). Detailed information can be found in Table S1. There is also a 20% presence of smokers in the control group, 9% in the mild group and none in the severe uncontrolled group, while 20% of the severe uncontrolled group are ex-smokers, as shown in Table 1. The presence of smokers in the control group is not a significant limitation, since the other groups also include smokers or ex-smokers, ensuring that any observed alterations in miRNA expression are not attributable to tobacco use. Additionally, regarding the sample size, although a larger cohort might be preferable, this cohort has been exhaustively studied and characterized, granting a high degree of homogeneity to this pilot study. The current sample size has been validated in previous works from our group and others [20,21,22], demonstrating its adequacy for the study’s objectives.

### 2.2. Severe Uncontrolled Allergic Asthmatic Patients Display a Unique miRNA Fingerprint in Serum

**Figure 1:**
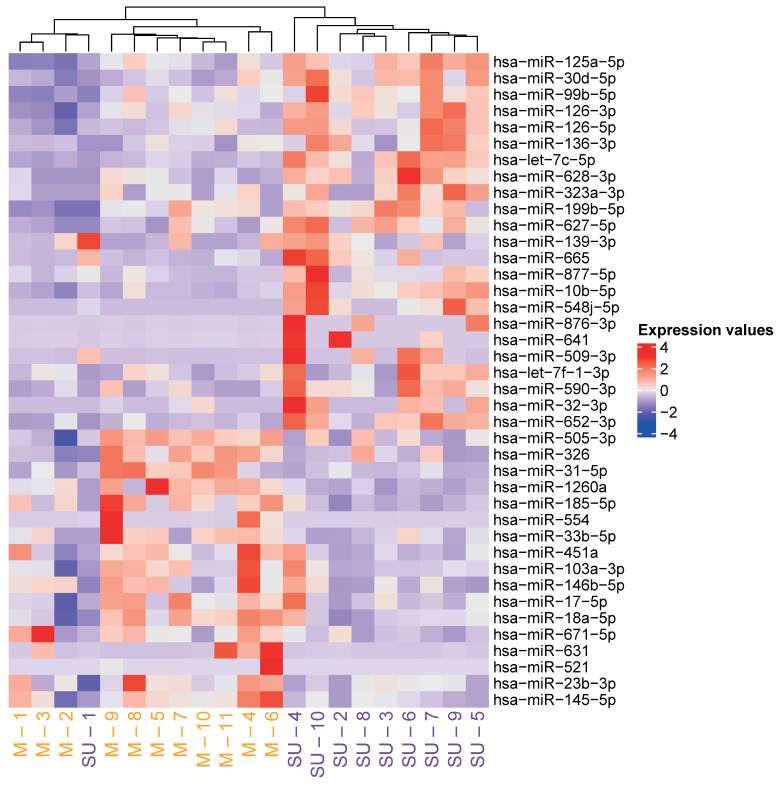
Analysis of DE miRNAs in severe uncontrolled and mild allergic asthmatic patients. Hierarchical clustering of Z-score normalized expression values of the 40 DE miRNAs between severe uncontrolled and mild allergic asthmatic patients. Yellow: mild group; purple: severe uncontrolled group.

As a mean to identify differentially expressed (DE) miRNAs associated with allergic asthma severity in this study, we performed a miRNA expression profiling analysis. From the detected miRNAs (*n* = 482 ± 113.24 miRNAs, Table S2), a total of 40 DE miRNAs (FDR (False Discovery Rate) *p*-adjusted value < 0.05) were found between the severe uncontrolled and mild groups resulting in 17 downregulated and 23 upregulated miRNAs in the severe uncontrolled patients (Tables S3 and S4, Supplementary Figure S1). The hierarchical clustering of expression values (Table S5) normalized by Z-score showed a clear grouping and differentiation between patient groups, confirming that severe uncontrolled allergic asthmatic patients display a unique miRNA fingerprint (Figure 1).

Thus, we have identified a miRNA signature that has the potential to differentiate between mild and severe uncontrolled allergic asthmatic phenotypes. 

### 2.3. Inflammation, Angiogenesis and Lipid Metabolism Signatures Are Enriched in Severe Allergic Asthmatic Patients

**Figure 2:**
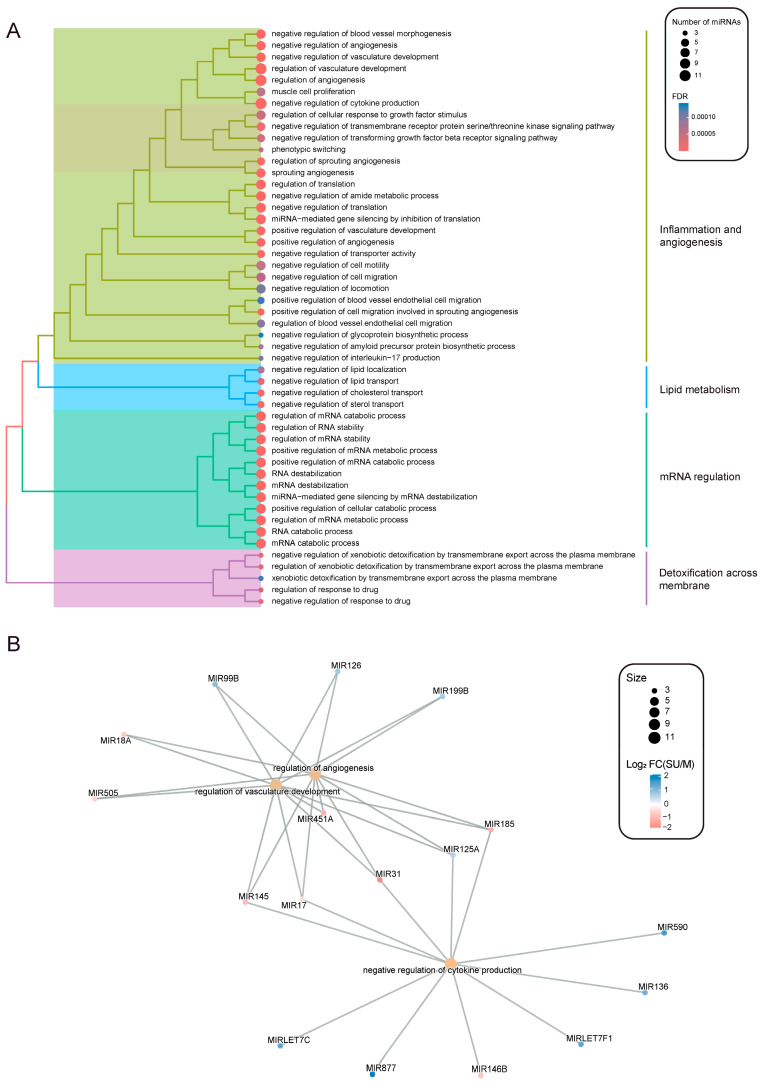
Enrichment analysis of biological processes from gene ontology terms using differentially expressed miRNAs between severe uncontrolled and mild allergic asthmatic patients. (**A**) Top 50 enriched processes are depicted (FDR < 0.05), where dot colors represent the *p*-adjusted values and sizes represent the number of miRNAs enriched per term. (**B**) Network plot showing those enriched terms with more than 10 miRNAs. miRNA expression (Log2(FC), severe uncontrolled vs. mild) is represented in color scale.

To identify biological functions affected by the DE miRNAs in the severe uncontrolled and mild groups, an enrichment analysis was performed based on the gene ontology (GO) biological process (BP) class. In total, we found 242 enriched BP terms (FDR < 0.05, Table S6). The top 50 (Figure 2A) are included in categories like inflammation and angiogenesis, lipid metabolism, mRNA regulation or detoxification across membrane.

Next, the biological processes with the highest number of miRNAs enriched (i.e., 11) were selected: ‘regulation of angiogenesis’, ‘regulation of vasculature development’ and ‘negative regulation of cytokine production’ (Figure 2A). The subsequent step was to explore the connection between these miRNAs and the three biological functions. We found that the same 11 miRNAs were implicated in both angiogenesis and vascular development signatures. However, six miRNAs were exclusively enriched in the ‘negative regulation of cytokine production’ process. Notably, five miRNAs (MIR145, MIR17, MIR131, MIR125A and MIR185) were shared between the vascular-related processes and the cytokine one, suggesting their potential as key regulators of the mechanisms underlying severity in allergic asthma.

Altogether, the identified DE miRNAs in severe uncontrolled allergic asthma are involved in relevant biological functions associated with different molecular signatures of the inflammatory response, angiogenesis and lipid metabolism.

### 2.4. 4 Severe Allergic Asthma miRNA Profile Correlates with Dysregulated Lipid Metabolism

**Figure 3:**
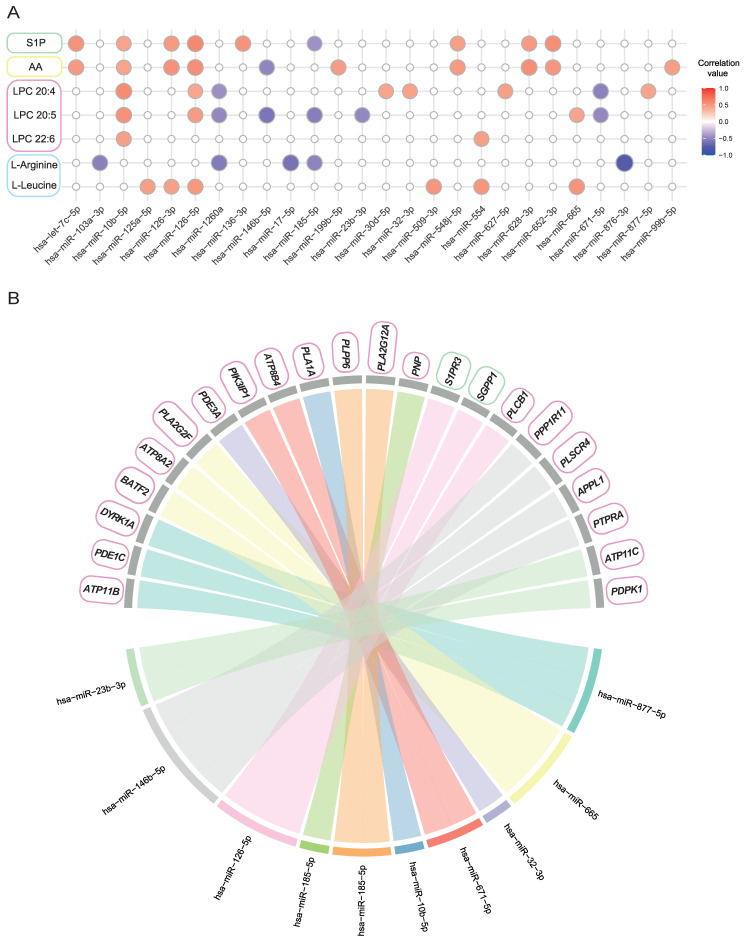
Correlations between differentially expressed miRNAs in severe uncontrolled and mild allergic asthmatic patients and several inflammatory-related metabolites, and predicted miRNA targets. (**A**) Correlation plot of 40 DE miRNAs and inflammatory-related metabolites. MiRNAs with no correlations are not included in the plot. Circles in blank are non-significant correlations (*p*-value > 0.05). (**B**) Predicted miRNAs’ targets (miRDB score > 80) linked to their correlated asthma-inflammatory-related metabolites. For both panels, color boxes: green, sphingolipids; yellow: fatty acids, pink: glicerophospholipids and blue, aminoacids.

Given the relevance of the metabolic and inflammatory signature categories obtained from the functional enrichment analysis and considering previously published data from our group on the metabolic changes associated with severe uncontrolled allergic asthmatic patients [20], we decided to analyze potential correlations between the serum levels of both, key metabolites (sphingosine-1-phosphate, arachidonic acid, L-arginine, L-leucine, lysophosphatidylcholine (LPC) 20:4, LPC 20:5 and LPC 22:6) and the 40 identified DE miRNAs. Our data showed significant correlations between 26 miRNAs and the analyzed metabolites (Figure 3A). In general, we found positive correlations between the severity-associated miRNAs and the metabolites, except for 7 miRNAs that were downregulated in the severe uncontrolled patients (Tables S5, S7 and S8).

Then, we determined the miRNA target genes associated with the correlated metabolites by using the miRDB database [23]. Surprisingly, we identified 10 DE miRNAs whose targets were protein-coding genes associated with the sphingosine-1-phosphate and lipid metabolic routes (Figure 3B, Table S9), most of them having multiple targets, such as miRNA hsa-miR-126-5p, whose predicted targets were sphingosine-1-phosphate receptor 3 (*S1PR3*, target score: 100), sphingosine-1-phosphate phosphatase 1 (*SGPP1*, target score: 85) and phospholipase C beta 1 (*PLCB1*, target score: 93). 

Thus, our results highlight that allergic asthmatic patients have altered levels of miRNAs that regulate target genes involved in the bioavailability of bioactive lipid mediators such as sphingosine-1-phosphate.

### 2.5. DE miRNAs Can Accurately Classify Allergic Asthmatic Patients According to Their Severity

**Figure 4:**
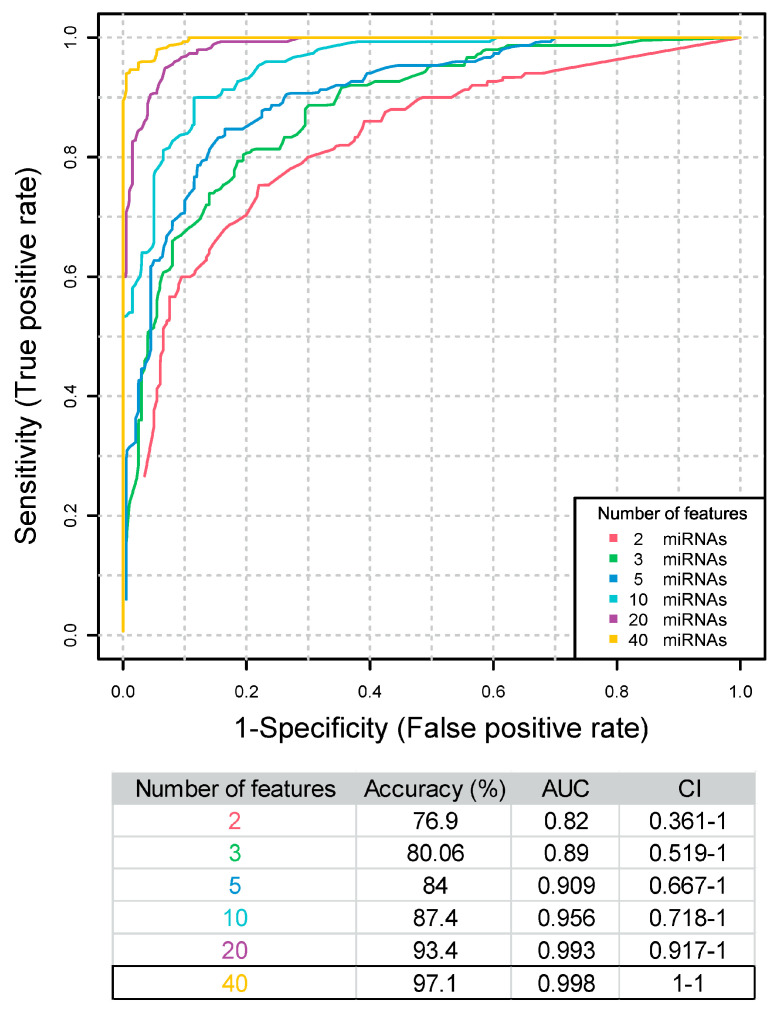
Random forest-based classifier. ROC curves of the generated models. Table showing accuracies, area under the curve (AUC) values and confidence intervals (CIs) of the trained models with different number of features. The best model is marked.

To know whether the set of 40 miRNAs can classify allergic asthma patients according to severity, we trained and evaluated the performance of a random forest-based classifier with cross-validation (input data in Tables S10 and S11). This resulted in the generation of six models that include different number of miRNAs determined by the algorithm. All the models presented high AUC values (from 0.82 to 0.998) and high accuracies (from 76,9% to 97.1%) (Figure 4). The best model was built using the 40 DE miRNAs with the highest AUC (0.998) and accuracy (97.1%). All miRNAs used by this model showed a selected frequency of 1, meaning that through the cross-validation step all the DE miRNAs were always selected to generate the evaluated models, suggesting they are key features for patient severity classification (Supplementary Figure S2A). With the best proposed model (Figure 4, yellow), a clear separation of the groups was achieved (Table S12, Supplementary Figure S2B).

### 2.6. Validation of miRNAs as Biomarkers for Severity Degree in Allergic Asthma

**Figure 5:**
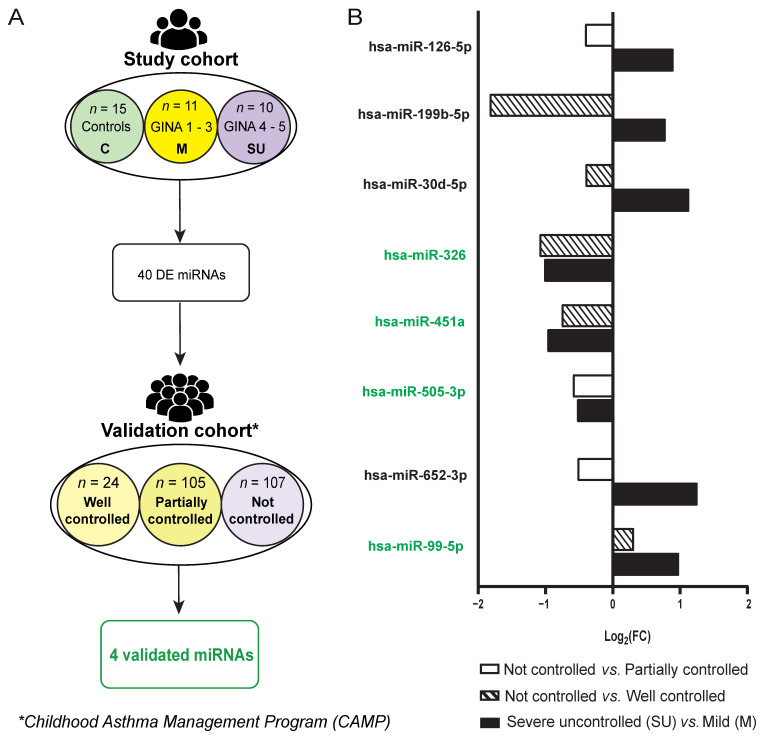
Differentially expressed miRNAs in validation cohort. (**A**) Workflow for biomarkers identification. (**B**) Bar plot of the relative miRNA expression values indicated by Log2(FC) of DE miRNAs in study cohort (severe uncontrolled (SU) vs. mild (M) comparative, black) and validation cohort (not controlled vs. well controlled comparative, striped; not controlled vs. partially controlled comparative, white). Validated miRNAs are written in green. * Childhood Asthma Management Program (CAMP) cohort.

Finally, we aimed to validate whether the miRNA severity fingerprint identified in our study was validated in other HDM-allergic asthmatic cohorts and could be associated with severity. With that purpose, we used the Childhood Asthma Management Program (CAMP) cohort, which has been exhaustively characterized [24,25]. Patients from the CAMP cohort with miRNA sequencing data were classified, according to GINA guidelines (steps 1–4) [8], into well controlled patients (*n* = 24), partially controlled patients (*n* = 105) and not controlled patients (*n* = 107) using symptom questionnaires (Figure 5A). It should be noted that this cohort does not include GINA step 5 patients, but severity status is used to generate subgroups based on GINA guidelines (steps 1–4) and therefore serves to validate our findings associated with severity (altered miRNAs in severe uncontrolled vs. mild group should also be altered when comparing different severity degrees).

We checked whether the 40 DE miRNAs in the study cohort were also DE in the validation cohort. From this set, 31 miRNAs had good quality sequencing data in the CAMP cohort (23 for not controlled vs. well controlled patients and 19 for not controlled vs. partially controlled). Considering those miRNAs with good quality, 8 of them were also DE in the validation cohort (Table S13). When comparing miRNA expression levels between the most extreme groups (not controlled vs. well controlled patients) inside the validation cohort, we found that 3 miRNAs (hsa-miR-99b-5p, hsa-miR-451a and hsa-miR-326) were also DE (*p*-value < 0.05) and displayed the same trend as in our study (Figure 5B). Moreover, hsa-miR-505-3p miRNA was significatively downregulated in a less extreme comparison (not controlled vs. partially controlled) in the validation cohort, as in the severe uncontrolled vs. mild comparison of our study. We could also observe that, in spite of being detected in the validation cohort, some DE miRNAs did not share the same trend between cohorts, such as hsa-miR-652-3p and hsa-miR-126-5p (Figure 5B). Strikingly, from the four inter-cohort-validated miRNAs, hsa-miR-99b-5p presented significant correlations with arachidonic acid (Figure 3A), a metabolite with high relevance in the inflammatory response taking place in allergic asthma.

Altogether, our data identified the hsa-miR-99b-5p, hsa-miR-451a, hsa-miR-326 and hsa-miR-505-3p inter-cohort-validated miRNAs as potential biomarkers to stratify allergic asthma severity.

## 3. Discussion

This work arises from the need of providing personalized treatment to allergic asthmatic patients. Through the analysis of miRNA patient profiles, we identified novel molecules and biological processes that reveal insights into the mechanisms underlying the severity of allergic asthma, offering valuable tools for patient stratification and the formulation of personalized medicine strategies.

We first worked in a study that included a cohort of HDM-allergic asthmatic patients from the Canary Islands that has been deeply characterized [20], giving reliability and reducing variability in the study. The Canary Islands’ conditions, with an almost tropical climate of high humidity and warm temperatures, lead to the increasing of mite exposure and sensitization prevalence to HDMs. These environments are linked to specific climatological conditions mainly occurring in islands where the prevalence of HDM allergy is very high, such as Singapore or New Zealand; therefore, although this study has been carried out in that specific region, the results can be extrapolated to other areas around the world [20]. 

The subjects involved in the study cohort were recruited by a unique and experienced clinical group, and the 21 allergic asthmatic patients were classified following GINA guidelines [8]. Remarkably, this cohort has been exhaustively studied and characterized, granting homogeneity to the study and, although the cohort under study may require a larger number of participants, the sample size has been proven to succeed in other works from our group and others [20,21,22]. Moreover, the fact that we have been able to validate the obtained results in the CAMP cohort reinforces these statements. These findings need to be validated in additional cohorts to enhance result generalizability. 

In order to identify miRNAs as severity biomarkers, we studied miRNA expression in serum, a non-invasive biological matrix used worldwide in clinical daily basis [10], rather than in bronchoalveolar lavage fluid (BALF), a more invasive, difficult to obtain sample [26]. Previous works have been mainly focused on studying miRNA expression differences in asthmatic patients vs. healthy subjects [19,27,28]. However, only a few took into consideration different degrees of severity; most of the altered miRNAs have been detected in other sample sources such as eosinophils, sputum or BALF [27].

We identified a signature of 40 DE miRNAs between severe uncontrolled and mild allergic asthmatic patients that allowed a clear classification of the patients according to their severity. Among them, some were previously reported to be differentially expressed in asthmatic patients compared to healthy subjects, not considering asthma severity as a factor. For instance, miR-33b-5p targets CCL2 [29] and was downregulated in the severe uncontrolled patients of the study cohort compared to the mild patients. CCL2 levels are increased in the blood of asthmatic patients compared with healthy controls, contributing to fibrocyte migration and to airway smooth muscle hyperplasia development in asthma [30]. In addition, periostin negative regulator miR-185-5p was found downregulated in severe uncontrolled patients, what is in line with reported miR-185-5p downregulation and higher periostin levels detected in asthmatic patients compared to healthy subjects [31]. Periostin is currently considered a biomarker in asthma diagnosis [32], although it is not a reliable severity degree indicator [33]. It might be reasonable to suggest that expression changes of miR-185-5p could add value to the use of periostin in the stratification of patients according to their severity. However, there are only a few studies that focus on miRNA expression differences between asthmatic patients of different severity. As previously reviewed [27], this is the case of miR-19a and let-7-a, found to be upregulated in bronchial epithelial cells [34] and downregulated in bronchial biopsies from severe vs. mild patients [35], respectively. No difference in the expression of these two miRNAs was detected in our study cohort, maybe because different biological matrixes were used. As we reflected, there is a lack of studies focusing on miRNA expression differences between asthma severity degrees in non-invasive samples such as serum. 

Interestingly, we corroborated that our signature of DE miRNAs is mainly involved in inflammation, angiogenesis, lipid metabolism and mRNA regulation. The enriched signatures are involved in different asthma events, such as an exacerbated immune response, airway remodeling and metabolic changes [5,7,20]. Thus, the alteration on the miRNA profile in these patients accurately reflects the complexity of allergic asthma and provides insights into its underlying mechanisms.

The correlations between the levels of miRNAs and severe uncontrolled allergic asthma-associated metabolites [20] in serum suggest that miRNAs might be triggering a potential regulation of metabolic and inflammatory pathways. One of the most significant altered signatures in severe uncontrolled patients was the sphingolipid signaling pathway, a metabolic route already known to participate in asthma susceptibility, initiation and exacerbation [36]. Alterations in sphingolipid levels have been previously reported in asthma [20], but their causes are still unknown. The sphingolipid signaling pathway plays a crucial role in asthma pathogenesis, influencing airway hyperresponsiveness, inflammation and remodeling [36]. Sphingolipids, particularly sphingosine-1-phosphate (S1P) and ceramide, are elevated in the airways of asthma patients and induce airway smooth muscle contraction. Moreover, plasma S1P levels have been associated with adult asthma disease control [37] and severity [38], while serum sphingolipid levels, particularly various ceramide species, have been linked to childhood asthma [39]. Interestingly, S1P has been identified as a potential biomarker for aspirin-exacerbated respiratory disease (AERD) [40], a severe asthma endotype. Remarkably, it has been described that environmental factors can significantly alter host cell sphingolipid metabolism, potentially contributing to asthma development and severity [36]. Animal studies have shown promising resultsin targeting sphingolipid species as a novel class of asthma therapeutics, though the results have been mixed and require further investigation [36]. Additionally, our research has identified several miRNAs that play a role in regulating the sphingolipid signaling pathway in asthma and, thus, they may serve as potential therapeutic targets. For instance, hsa-miR-126-5p’s predicted target genes are related to sphingosine-1-phosphate metabolism and receptors (SGPP1 and S1PR3) and phospholipid metabolism (PLCB1). Previous work demonstrates that miRNAs alter the expression of Orosomucoid like 3 (ORMDL3) [41], which regulates the activity of serine palmitoyltransferase (SPT), the first and rate-limiting enzyme for sphingolipid biosynthesis in cells [42]. In fact, ORMDL3 is a major factor in asthma development [41]. As a whole, this association clearly implicates these miRNAs in the mechanisms underlying allergic asthma severity.

Future studies should focus on developing miRNA-based therapies that can fine-tune sphingolipid signaling, potentially offering new avenues for asthma treatment and management.

Furthermore, the found set of 40 miRNAs accurately classified the severity of allergic asthma. Using a random forest-based classifier, we trained several models using a different number of features (miRNAs). Every miRNA in our best classifier has been selected thorough cross-validation steps, ensuring that each miRNA robustly contributes to the model’s unparalleled performance. This selection process guarantees that our classifier provides clear and definitive group separations, offering precision in diagnosing and understanding allergic asthma severity. 

Once the miRNAs associated with severity were identified through comparison between extreme phenotypes, the next step was to validate these markers in a population of allergic asthma with a more homogeneous profile, where the highest severity step was GINA 4. In this case, differential expression of 4 out of the 40 DE miRNAs in the most extreme group comparisons (hsa-miR-505-3p, hsa-miR-99b-5p, hsa-miR-451a and hsa-miR-326) was validated. It is worth noting that the validation cohort includes patients from GINA step 4 instead of 5 and is a pediatric cohort, while the study cohort includes only adult severe patients that belong to GINA step 5, mainly uncontrolled patients. Altogether, the validation analysis demonstrates that this set of miRNAs are applicable to other cohorts regardless of the individuals’ age and the geographical location, only depending on severity status. Moreover, the miRNAs that were not validated here are not excluded from being validated in another cohort of adults in GINA step 5, so this point remains outstanding for future research. 

Regarding the validated miRNAs, three of them (hsa-miR-99b-5p, hsa-miR-505-3p and hsa-miR-451a) are linked to inflammation and angiogenesis. Notably, our data showed that the overexpression of hsa-miR-99b-5p correlated with high levels of arachidonic acid in severe uncontrolled allergic asthmatic patients. Arachidonic acid regulates the function of many immune cell types such as eosinophils, neutrophils, monocytes, macrophages, dendritic cells, T cells and B cells by affecting transcription factor activation and gene expression [43]. Moreover, arachidonic acid precursors have been found to be increased in severe allergic disease [20], participating in platelet functionality alteration in inflammation [44]. 

Altogether, we identified a miRNA signature in severe uncontrolled allergic asthmatic patients associated with key inflammatory biological routes and alterations in lipid metabolism. These findings provide novel information for patient stratification and unravel new mechanisms complicit in the severity of allergic asthma.

## 4. Materials and Methods

### 4.1. Patients

For this study, 36 individuals (Table 1) were recruited by the Allergy Service at Hospital Universitario de Gran Canaria Dr. Negrín (Las Palmas de Gran Canaria, Spain), as previously described [20] and detailed in the supplementary data. The project was approved by the Ethics Committee from the hospital, and all subjects signed an informed consent agreement. 

Fifteen non-allergic and non-asthmatic subjects (classified according to their clinical history) were used as controls to normalize the RT-qPCR results. The remaining subjects were allergic asthmatic patients, and were stratified by severity, according to GINA guidelines (Global Initiative for Asthma [8]) and Test of Adherence to Inhalers (TAI), in mild (*n* = 11, GINA 1–3, controlled with conventional available treatments) and severe uncontrolled (*n* = 10, GINA 4–5, poor symptom control and/or frequent (≥2, requiring OCS) or severe (≥2, requiring hospitalization) exacerbations even with good adherence to the treatment) groups. Nineteen patients (90.5%) had a positive skin prick test to HDMs. Detailed information about subjects’ clinical characteristics can be found in the supplementary data (Table S1).

### 4.2. miRNAs Extraction and Expression Quantification

The miRCURY LNA miRNA miRNome PCR (Quiagen) kit was used for miRNA extraction and cDNA synthesis following the provided instructions. To quantify miRNAs’ expression, the RT-qPCR technique was applied using SYBR Green as a fluorescent calibration marker and ROX as a passive reference dye. The plates used were the miRCURY LNA miRNA miRNome PCR Human panel I+II. The exogen controls used for controlling the extraction process and qPCR reaction effectiveness were: UniSp2, UniSp4, UniSp5, UniSP6 and UniSp3 IPC. The endogenous controls present on plate I (SNORD84B and SNORD99A) are not suitable for use in serum samples; therefore, the GME (Global Mean Expression [45]) method (explained below) was used instead. The equipment used was the 7900HT Fast Real-Time PCR System with a 384-Well Block Module (Thermo-Fisher, Waltham, MA, USA).

Data were analyzed by SDS 2.4 7900HT Fast Real-Time PCR System software (Thermo-Fisher), and RQ-Manager 1.2.1 was used to generate amplification results data. 

All scripts were developed in R 4.0.3 [46] and are available in the following GitHub repository: https://github.com/Andrea290799/MicroRNA-profiling-of-severe-uncontrolled-allergic-asthmatic-patients (accessed on 29 August 2024). Data were pre-processed and normalized with miRNAs_normalization.R script (details about data pre-processing and inter-plate normalization can be found in the supplementary data). The 2^−ΔΔCt^ method [47] was applied for intra-plate normalization. The GME value was used as control in ΔCt normalization. This value results from obtaining the mean expression value of the entire plate miRNAs. Further details about data pre-processing, normalization and analysis can be found in the Supplementary Data. 

### 4.3. Statistics

Clinical characteristics were compared between the three groups. Quantitative variables were analyzed by a Shapiro–Wilk test for assessing the normality of the data. For normally distributed data, an ANOVA test was applied. Otherwise, a Kruskal–Wallis with Dunn’s post hoc was applied. The Mann–Whitney U test was used for assessing differences between two groups in cases of non-normal data. Association between binary variables was assessed using Fisher’s exact test. We considered a *p*-value threshold of 0.05 for significant results. 

For assessing correlations between severe uncontrolled allergic asthma-related (and previously described) metabolites with DE miRNAs, we used the Pearson test in the case of normally distributed data and Spearman test in the case of not normally distributed data.

Different statistical methods were applied for determining miRNA differential expression between the groups, by using miRNAs_diff_exp.R script. Each miRNA population was studied independently to assess if it was normally distributed (shapiro.test function) and homoscedastic (leveneTest function). For normally distributed and homoscedastic populations, an ANOVA (parametric, aov function) and its associated post hoc (T-test, pairwise.t.test function) were applied. Otherwise, a Kruskal–Wallis (non-parametric, kruskal.test function) and its related post hoc (Wilcoxon test, pairwise.wilcox.test function) were applied. Benjamini–Hochberg *p*-value correction for multiple testing was applied, considering FDR < 0.05 for significant results. 

### 4.4. Functional Enrichment Analysis

To perform an enrichment analysis of the 40 DE miRNAs, the R package clusterProfiler [48,49] was used (script clusterProfiler_enrichment-analysis.R). Only enriched terms with a Benjamini–Hochberg *p*-adjusted value (FDR) < 0.05 were considered. Previously, miRNA names needed to be transformed into symbols using the AnnotationDbi [50] and limma [51] R packages in order perform this analysis. 

### 4.5. Random Forest-Based Classifier

The MetaboAnalystR [52] R package was used for classifier modeling and performance evaluation through ROC curves (script Classifier_MetaboAnalystR.R). With random forest as the classification method, the RandomForest built-in as feature ranking method and Monte Carlo cross-validation were used. Missing values were replaced with 1/5 of the minimum positive values of individual features. Pre- and post-imputation data can be found in the Supplementary Data, Tables S9 and S10. 

### 4.6. miRNA Validation Analysis in CAMP (Childhood Asthma Management Program) Cohort

A different cohort from Brigham and Women’s Hospital and Harvard Medical School was used for the validation analysis. CAMP is a multi-center, randomized, double-blinded clinical trial of inhaled corticosteroids in 1041 children aged 5 to 12 years with mild-to-moderate persistent asthma who were followed for 4 years. We selected the 236 patients (well controlled patients (*n* = 24), partially controlled patients (*n* = 105) and not controlled patients (*n* = 107) from this cohort that had serum miRNA sequencing data available and a positive skin prick test result for HDMs [53] (validation cohort). Patient stratification followed GINA guidelines [8] about the 3 ordinal levels of asthma symptom control: well controlled, partially controlled and not controlled. The participants were asked about their history in the past year at enrolment, including their symptoms’ frequency and types of medication. These data were compiled into GINA scores. The miRNA data are from baseline, before any trial medication was administered. 

### 4.7. Data Analysis of miRNA Validation in CAMP Cohort

We tested whether the found DE miRNAs present in this study cohort were also differentially expressed in the patients of the CAMP cohort. The DESeq2 R package was used to normalize and analyze the sequencing data. MiRNAs were considered differentially expressed if *p*-value < 0.05 (Table S10).

## 5. Conclusions

Altogether, we identified a specific miRNA profile associated with severe uncontrolled allergic asthmatic patients, which is linked to key inflammatory biological pathways and changes in lipid metabolism. This profile is able to accurately classify allergic asthmatic patients according to their severity. These findings offer new insights for patient stratification and reveal novel mechanisms underlying the severity of allergic asthma.

## Figures and Tables

**Table 1:** Clinical information about the subjects.

	Control	Mild	Severe Uncontrolled
*n*	15	11	10
Age (years)	37.4 ± 13.95 *	36 ± 6.53 *	50.9 ± 10.62
Onset (years)	-	14.55 ± 10.21	10.7 ± 6.11
Gender (%F/%M)	60/40	63.64/36.36	60/40
BMI	28.71 ± 5.65	27.52 ± 5.80	28.35 ± 4.00
Smoker (%)	20	9.09	0
Ex-smoker (%)	0	0	20
Total IgE (U)	-	551.09 ± 733.8	462.1 ± 587.92
AC (%)	0	0 *	60
AH (%)	6.67	100 *	30
LTRA (%)	0	18 *	90
ICS/LABA (%)	0	90.91	100
OCS (%)	0	0	10
TCS (%)	6.67	100 *	30
SABA (%)	0	9.09 *	100
T (%)	0	0	10
*D. pteronyssinus* (%)	0	90.91	80
*D. farinae* (%)	0	90.91	80
*L. destructor* (%)	0	54.55	50
*B. tropicalis* (%)	0	81.82	40
*A. siro* (%)	0	36.36	10
*T. putrescentiae* (%)	0	45.45	50

Abbreviations: AC, anticholinergic; AH, antihistaminic; BMI, Body Mass Index; ICS/LABA, inhaled corticosteroid combined with long-acting beta-adrenoceptor agonist; LTRA, antileukotriene; OCS, oral corticosteroid; SABA, short-acting beta-adrenoceptor agonist; T, theophylline; TCS, topical corticosteroid; U, ISAC units. * *p* < 0.05 against severe uncontrolled.

## Data Availability

The original source code presented in the study are openly available in GitHub at https://github.com/Andrea290799/MicroRNA-profiling-of-severe-uncontrolled-allergic-asthmatic-patients (accessed on 29 August 2024).

## Supplementary Materials

The following supporting information can be downloaded at: https://www.mdpi.com/article/10.3390/ijms25179425/s1. References [20,23,37,38,54,55,56,57,58,59,60,61,62,63,64,65] are cited in Supplementary Materials.
