# Supplementary material for: Specific microRNA Profile Associated with Inflammation and Lipid Metabolism for Stratifying Allergic Asthma Severity
Source: Int J Mol Sci. 2024 Aug 30;25(17):9425. doi: 10.3390/ijms25179425 (PMC11394998; doi:10.3390/ijms25179425)
Supplement: Supplementary file 1 [file ijms-25-09425-s001.zip › Supp_Figure S1.pdf]

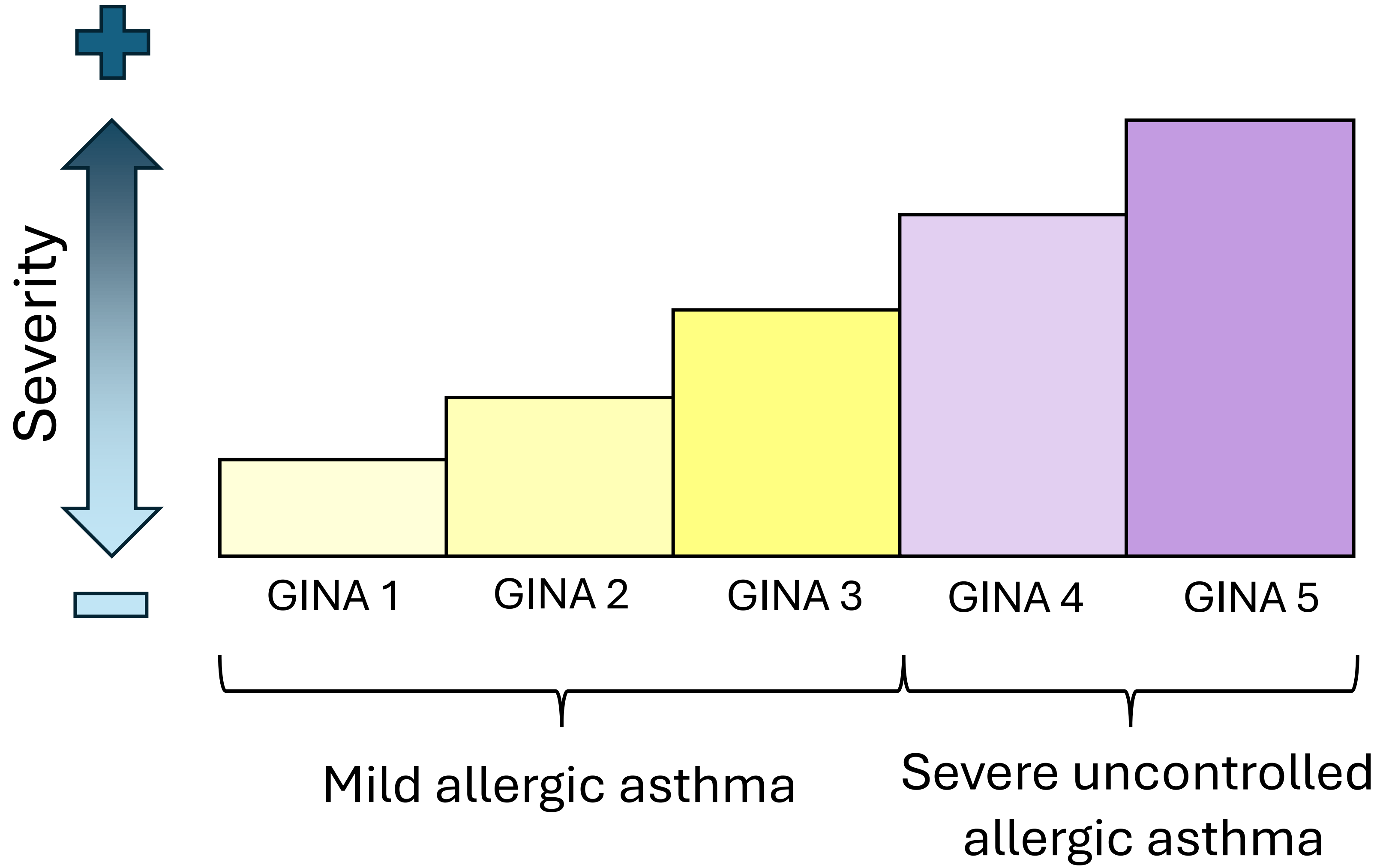

Upregulated miRNAs in severe uncontrolled group comparing to mild group**:**

hsa-miR-548j-5p, hsa-miR-10b-5p, hsa-miR-665, hsa-miR-30d-5p, hsa-miR-323a-3p, hsa-miR-877-5p, hsa-miR-125a-5p, hsa-miR-126-5p, hsa-miR-627-5p, hsa-miR-876-3p, hsa-miR-641, hsa-miR-99b-5p, hsa-miR-509-3p, hsa-let-7c-5p, hsa-let-7f-1-3p, hsa-miR-628-3p, hsa-miR-32-3p, hsa-miR-126-3p, hsa-miR-199b-5p, hsa-miR-590-3p, hsa-miR-136-3p, hsa-miR-652-3p, hsa-miR-139-3p

Upregulated miRNAs in mild group comparing to severe uncontrolled group**:**

hsa-miR-185-5p, hsa-miR-145-5p, hsa-miR-1260a, hsa-miR-33b-5p, hsa-miR-326, hsa-miR-18a-5p, hsa-miR-671-5p, hsa-miR-505-3p, hsa-miR-554, hsa-miR-451a, hsa-miR-31-5p, hsa-miR-23b-3p, hsa-miR-103a-3p, hsa-miR-631, hsa-miR-17-5p, hsa-miR-146b-5p, hsa-miR-521
