# Supplementary material for: Specific microRNA Profile Associated with Inflammation and Lipid Metabolism for Stratifying Allergic Asthma Severity
Source: Int J Mol Sci. 2024 Aug 30;25(17):9425. doi: 10.3390/ijms25179425 (PMC11394998; doi:10.3390/ijms25179425)
Supplement: Supplementary file 1 [file ijms-25-09425-s001.zip › Supplementary_data.pdf]

Supplementary data

Title: Specific microRNA profile associated with inflammation and lipid metabolism for stratifying allergic asthma severity

Authors: Andrea Escolar-Peña, María Isabel Delgado-Dolset, Carmela Pablo-Torres, Carlos Tarin, Leticia Mera-Berriatua, María del Pilar Cuesta Apausa, Heleia González Cuervo, Rinku Sharma, Alvin T. Kho, Kelan G Tantisira, Michael J McGeachie, Rocio Rebollido-Rios, Domingo Barber, Teresa Carrilo, Elena Izquierdo*, María M Escribese*.

*These authors equally contributed

Patient profiling and clinical characteristics

Patients were tested using standardized extracts of the following HDM: *Dermatophagoides pteronyssinus, Dermatophagoides farinae, Blomia tropicalis, Acarus siro, Lepidoglypus destructor* and *Tyrophagus putrescentiae*. Skin prick test was considered as positive when the larger diameter of the wheal was at least 3 mm bigger than that of the negative control.

Regarding patient stratification, patients were considered severe if they needed treatment from steps 4-5 of the GINA, or 5-6 of the GEMA guidelines (ICS/LABA in high doses + LTRA + theophylline) and/or had to take systemic corticosteroids in the previous year. The uncontrolled patients had approximately 5 exacerbations per year.

Asthma control was assessed using both Asthma Control Test (ACT) and objective factors such as symptom control, cease of exacerbations in the prior

year, reduction in bronchodilator usage, etc. Inclusion criteria for the controlled groups was that patients had not suffered any exacerbations for a year prior to their enrolment in this study.

Sample collection and processing

Whole blood was collected and incubated with a clotting agent using Vacutainer SST II tubes (BD). Samples were placed at room temperature for 30 min and then centrifuged at 2000 x g for 10 min. Serum was collected and stored at -80º C.

MiRNAs expression quantification

For data pre-processing, undetermined values were replaced with either “40” (maximum number of PCR cycles) or “NA” (not available data) following specific criteria. If all Ct values of a particular miRNA were undetermined in the 3 groups, that miRNA was taken out of the analysis. If all Ct values of a miRNA were undetermined in one or two groups, the undetermined values were substituted with 40. If there were some undetermined Ct values all in the same group, they were replaced by 40 if ΔRn (magnitude of the signal generated by the given set of PCR conditions) > 0.01 (10 times smaller than the smallest ΔRn experimentally observed); otherwise, they were considered “NA”.

For inter-plate normalization we followed the next criteria: if any UniSp3 IPC value deviated 0.5 Ct from the other two in a particular plate, the plate was deleted. Normalization process consisted in calculating the mean value of all UniSp3 of each plate (PUME, Plate UniSp3 Mean Expression). Then, the mean value of

these PUMEs was calculated (GUME, Global UniSp3 Mean Expression). Difference between PUME and GUME was the normalization factor of each plate. The Ct value of each miRNA on each plate was adjusted by subtracting the corresponding normalization factor of the plate.

Data analysis and representation

A hierarchical clustering of the DE miRNAs of each patient was performed with R programming language (Heatmap.R). The resulting heatmap was plotted with Heatmap function, from ComplexHeatmap [1,2] R package. Expression data ($2^{-\Delta\Delta Ct}$ values) were normalized by Z-score before the clustering and representation. NA values were considered 0, as the algorithm cannot work with NA values; this transformation is backed up in the idea that there is no ΔΔCt able to meet the following expression: $2^{-\Delta\Delta Ct} = 0$ (Table S5).

For the treeplot of enrichment analysis results, only top 50 signatures (order based on FDR) were represented; hclust function was used for clustering generation (4 clusters and "centroid" as clustering method) and cluster names were rewritten, as the R function labels subtrees using high-frequency words. For the signature-miRNA network representation, only signatures with more than 10 miRNAs were selected. Both the treeplot and the cnetplot were obtained with clusterProfiler_enrichment-analysis.R script.

A correlation matrix between the 40 DE miRNAs' normalized expression values and the abundance of several inflammatory-related metabolites' that we had previously described to be characteristic of severe uncontrolled allergic asthmatic patients [3] was calculated in script Correlations.R. Pearson correlation test was

applied when both miRNA and metabolite populations were gaussian; otherwise, Spearman correlation test was chosen. For plotting the correlation map, ggcorrplot function from ggcorrplot [4] R package was used. Then, we used miRDB [5] online database to search DE miRNAs' predicted targets, restricting the search to those targets related to correlated metabolites for each DE miRNA. Results were represented with a chordplot made with circlize [6] R package (Chordplot.R script).

Metabolomic analysis

Metabolomic analysis was performed as previously described in [3]. Briefly, serum samples were prepared and measured in batches, in a randomized order using a liquid chromatography coupled to mass spectrometry (LC-MS) with a quadrupole-time of flight (Q-TOF) analyser (Agilent series 6520). The experiment, as previously described [7] was measured in electrospray ionization in positive and negative modes (ESI+ and ESI−, respectively). A quality control (QC) sample was prepared by mixing equal volumes of a representative set of samples and was analysed throughout the analyses to ensure instrumental reproducibility. Metabolite annotation was performed using CEU Mass Mediator 3.0 online data base [8] and confirmed through tandem mass experiments (MS/MS) with a fragmentation energy of 20 eV.

Metabolites' selection criterion is based on main findings in Delgado-Dolset *et al.* [3]. It has been previously published that the LPCs have been associated to arachidonic acid release in human derived monocytes [9], as well as being reported in different functional enrichment analyses in asthma, together with their

related pathways [10–12]; it has also proved that L-arginine and L-leucine are related to nitric oxide (NO) pathway [13] and to the activation of mTORC1 pathways responsible for T cells activation, proliferation and differentiation [14], respectively; remarkably, the contribution to asthma pathophysiology of sphingosine-1-phosphate [13,15] and arachidonic acid is widely known [16].

References

1. Gu Z. Complex heatmap visualization. iMeta. 2022;1(3).
2. Gu Z, Eils R, Schlesner M. Complex heatmaps reveal patterns and correlations in multidimensional genomic data. Bioinformatics. 2016;32(18):2847–9.
3. Delgado-Dolset MI, Obeso D, Rodríguez-Coira J, Tarin C, Tan G, Cumplido JA, et al. Understanding uncontrolled severe allergic asthma by integration of omic and clinical data. Allergy. 2022;77(6):1772–85.
4. Alboukadel Kassambara. ggcorrplot: Visualization of a Correlation Matrix using ‘ggplot2’. https://CRAN.R-project.org/package=ggcorrplot. 2023.
5. Chen Y, Wang X. miRDB: an online database for prediction of functional microRNA targets. Nucleic Acids Res. 2020;48(D1):D127–31.
6. Gu Z, Gu L, Eils R, Schlesner M, Brors B. *circlize* implements and enhances circular visualization in R. Bioinformatics. 2014;30(19):2811–2.
7. Rodríguez-Coira J, Delgado-Dolset M, Obeso D, Dolores-Hernández M, Quintás G, Angulo S, et al. Troubleshooting in Large-Scale LC-ToF-MS

Metabolomics Analysis: Solving Complex Issues in Big Cohorts. Metabolites. 2019;9(11):247.

8. Gil-de-la-Fuente A, Godzien J, Saugar S, Garcia-Carmona R, Badran H, Wishart DS, et al. CEU Mass Mediator 3.0: A Metabolite Annotation Tool. J Proteome Res. 2019;18(2):797–802.

9. Oestvang J, Anthonsen MW, Johansen B. LysoPC and PAF Trigger Arachidonic Acid Release by Divergent Signaling Mechanisms in Monocytes. J Lipids. 2011;2011:1–11.

10. Sokolowska M, Chen LY, Liu Y, Martinez-Anton A, Logun C, Alsaaty S, et al. Dysregulation of lipidomic profile and antiviral immunity in response to hyaluronan in patients with severe asthma. Journal of Allergy and Clinical Immunology. 2017;139(4):1379–83.

11. Nie X, Wei J, Hao Y, Tao J, Li Y, Liu M, et al. Consistent Biomarkers and Related Pathogenesis Underlying Asthma Revealed by Systems Biology Approach. Int J Mol Sci. 2019;20(16):4037.

12. McGeachie MJ, Dahlin A, Qiu W, Croteau-Chonka DC, Savage J, Wu AC, et al. The metabolomics of asthma control: a promising link between genetics and disease. Immun Inflamm Dis. 2015;3(3):224–38.

13. Reinke SN, Gallart-Ayala H, Gómez C, Checa A, Fauland A, Naz S, et al. Metabolomics analysis identifies different metabotypes of asthma severity. European Respiratory Journal. 2017;49(3):1601740.

14. Ananieva EA, Powell JD, Hutson SM. Leucine Metabolism in T Cell Activation: mTOR Signaling and Beyond. Advances in Nutrition. 2016;7(4):798S-805S.

15. Petrache I, Berdyshev E V. Ceramide Signaling and Metabolism in Pathophysiological States of the Lung. Annu Rev Physiol. 2016;78(1):463–80.

16. Luo Y, Jin M, Lou L, Yang S, Li C, Li X, et al. Role of arachidonic acid lipoxygenase pathway in Asthma. Prostaglandins Other Lipid Mediat. 2022;158:106609.
