# Supplementary material for: Specific microRNA Profile Associated with Inflammation and Lipid Metabolism for Stratifying Allergic Asthma Severity
Source: Int J Mol Sci. 2024 Aug 30;25(17):9425. doi: 10.3390/ijms25179425 (PMC11394998; doi:10.3390/ijms25179425)
Supplement: Supplementary file 1 [file ijms-25-09425-s001.zip › Supplementary_Figures_captions.pdf]

Supplementary Figure S1. **Stratification of patient types and miRNAs associated.** GINA steps height represents severity degree of allergic asthma.

Supplementary Figure S2. Random Forest-based classifier metrics. **(A)** Frequency of selection of the miRNAs in the cross-validation process. Expression of the miRNAs is marked in red (over-expression) or blue (under-expression) comparing both groups of patients (M: mild and SU: severe uncontrolled). **(B)** Predicted class probabilities of the samples (M: mild and SU: severe uncontrolled).
