# Supplementary figures and images for: Specific microRNA Profile Associated with Inflammation and Lipid Metabolism for Stratifying Allergic Asthma Severity
Source: Int J Mol Sci. 2024 Aug 30;25(17):9425. doi: 10.3390/ijms25179425 (PMC11394998; doi:10.3390/ijms25179425)

A

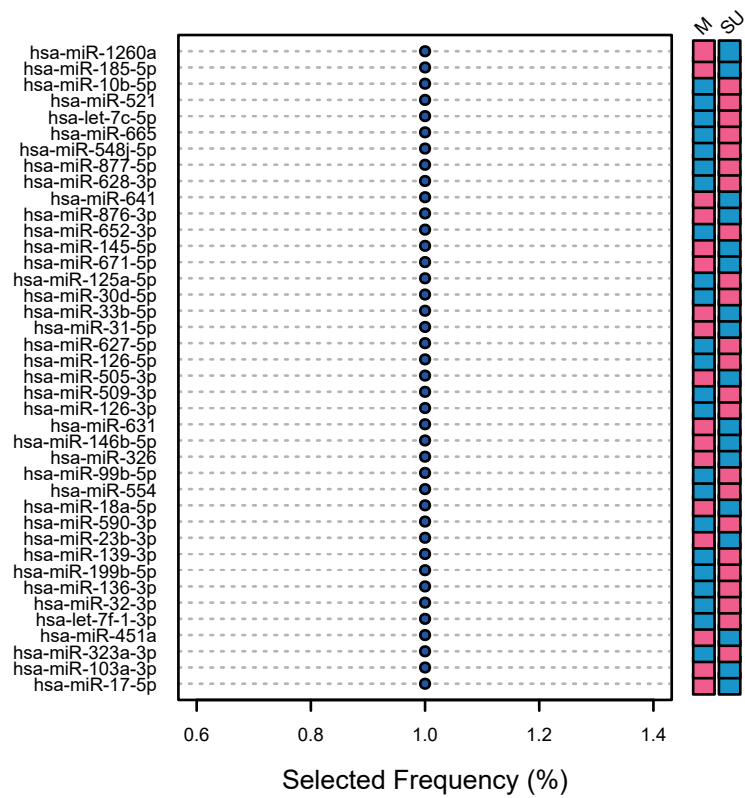

B

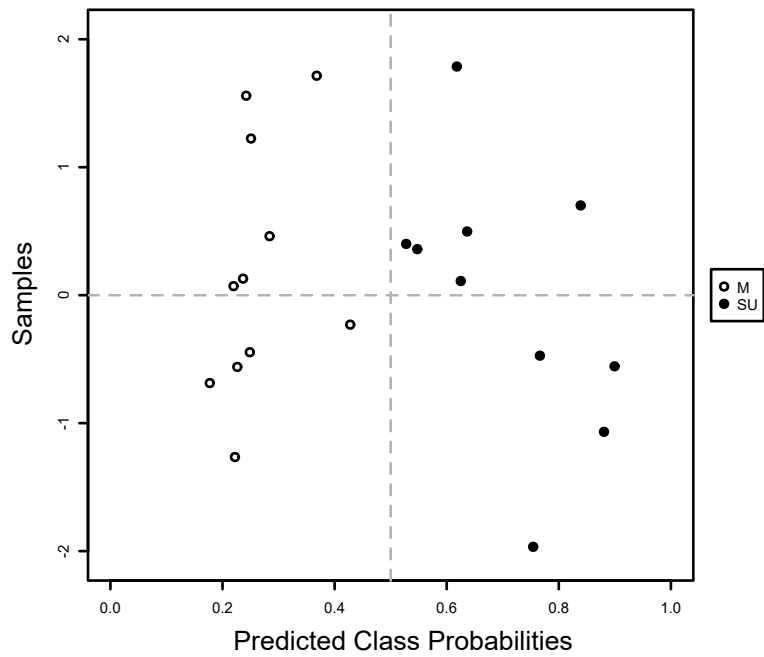

Supplement: Supplementary file 1 [file ijms-25-09425-s001.zip › Supp_Figure S2.pdf]
